# 鉴定晚期肺腺癌*EGFR*-T790M耐药基因突变候选生物标志物

**DOI:** 10.3779/j.issn.1009-3419.2020.104.20

**Published:** 2020-11-20

**Authors:** 丽鹃 陈, 莉 单, 婷婷 俞

**Affiliations:** 830000 乌鲁木齐，新疆医科大学附属肿瘤医院肺内科一病区 Department of Pulmonary Medicine Ward 1, Affiliated Tumor Hospital of Xinjiang Medical University, Urumqi 830000, China

**Keywords:** 晚期肺腺癌, EGFR-T790M, 蛋白组学, 差异性蛋白, Advanced lung adenocarcinoma, EGFR-T790M, Proteomics, Differentail proteins

## Abstract

**背景与目的:**

奥希替尼（Osimertinib）是美国食品和药物管理局（Food and Drug Administration, FDA）批准用于携带表皮生长因子受体（epidermal growth factor receptor, *EGFR*）-T790M突变的晚期非小细胞肺癌（non-small cell lung cancer, NSCLC）患者的药物，用药前需行*EGFR*-T790M检测。不少患者因进展病灶隐匿或体弱无法进行组织活检错过Osimertinib治疗，本研究希望能从血清中筛查出预测*EGFR*-T790M耐药突变相关蛋白，为临床用药提供帮助。本研究旨在探索*EGFR*-T790M耐药基因相关蛋白，为临床用药提供帮助。

**方法:**

本研究纳入口服易瑞沙晚期肺腺癌患者36例，在疾病进展后行组织活检，使用ARMS方法检测出*EGFR*-T790M突变组患者18例，非*EGFR*-T790M突变组18例。收集耐药患者血清，采用同位素标记相对和绝对定量标记结合二维液相色谱串联质谱蛋白组学技术筛选与*EGFR*-T790M耐药相关蛋白。

**结果:**

筛出17种差异性蛋白，与*EGFR*-T790M基因突变相关上调蛋白6种，下调蛋白11种，主要参与31种生物过程，7种细胞组分，26种分子功能；反应途径中共鉴定出12种富集途径，其中富集指数最高的是凝血级联途径。

**结论:**

发现与*EGFR*-T790M耐药相关蛋白共17种，参与凝血级联途径蛋白有望成为预测*EGFR*-T790M耐药突变相关的生物标志物。

## Abstract (en)

**Background and objective:**

Osimertinib is approved by Food and Drug Administration for patients with advanced non-small cell lung cancer carrying *EGFR*-T790M mutations. Osimertinib therapy was missed in many patients who were unable to perform biopsy due to occult lesion progression or weak body. In this study. We hope that some proteins associated with predicting *EGFR*-T790M resistance could be screened from the serum to provide help for clinical medication. The aim of this study is to explore the protein associated with *EGFR*-T790M drug resistance gene and provide help for clinical medication.

**Methods:**

In this study, 36 patients with advanced lung adenocarcinoma treated by gefitinib were included. After the disease progression of the patients, biopsy was performed. 18 patients in the *EGFR*-T790M mutation group and 18 patients in the non-*EGFR*-T790M mutation group were detected by the ARMS method. Serum of patients with drug resistance was collected, and proteins related to *EGFR*-T790M resistance were screened by isotopic marker relative and absolute quantitative marker combined with two-dimensional liquid chromatography tandem mass spectrometry proteomics technology.

**Results:**

Seventeen different proteins were screened out, including 6 up-regulated proteins and 11 down-regulated proteins associated with *EGFR*-T790M gene mutation, which were mainly involved in 31 biological processes, 7 cell components and 26 molecular functions. Twelve enrichment pathways were identified, among which the highest enrichment index was the coagulation cascade pathway.

**Conclusion:**

Seventeen proteins associated with *EGFR*-T790M resistance were found, and proteins involved in the coagulation cascade pathway are expected to be biomarkers associated with predicting *EGFR*-T790M resistance mutations.

表皮生长因子受体（epidermal growth factor receptor, *EGFR*）是非小细胞肺癌（non-small cell lung cancer, NSCLC）中最常见的驱动基因，其中*EGFR* 19del和*EGFR* 21L858R是最常见的基因突变类型，具有以上基因突变的晚期NSCLC可使用第一代EGFR酪氨酸激酶抑制剂（EGFR-tyrosine kinase inhibitors, EGFR-TKIs）：易瑞沙或者特罗凯进行抗肿瘤治疗。但患者在用药后的9个月-13个月都不可避免的出现了耐药^[1]^。*EGFR*-T790M突变是一代EGFR-TKI最常见的耐药机制，而IL-6/JAK1/STAT3、PI3K/Akt信号通路激活、*Kras*突变、Fox M1上调等为其他常见耐药机制^[2]^。第三代EGFR-TKI Osimertinib被批准用于携带*EGFR*-T790M耐药基因突变患者，极大地延长了晚期NSCLC患者生存期。但不少患者因进展病灶隐匿活检困难，或因其他耐药突变而无法使用Osimertinib。根据最新FLAURA研究结果^[3]^，Osimertinib一线用于*EGFR*突变晚期NSCLC患者中，中位总生存期优于续惯EGFR-TKI治疗患者。尽管奥西替尼在*EGFR*-T790M突变的NSNCL取得卓越疗效，而那些因为病灶隐匿、年老体弱或者体液检测假阴性的患者依然无法从中获益^[4-8]^。本研究旨在使用蛋白组学的方法鉴定出与*EGFR*-T790M基因突变相关的生物标志物，为无法二次活检或体液检测假阴性患者提供帮助。而蛋白组学技术具有很高的灵敏度和可重复性，已被广泛用于研究药物对恶性肿瘤发生发展机制的影响。

## 1 资料与方法

### 1.1 临床资料

选取2018年1月-2018年12月就诊于新疆医科大学附属肿瘤医院具有*EGFR* 19缺失或*EGFR* 21L858R基因突变晚期肺腺癌患者36例，根据病情需要口服易瑞沙治疗，采用高分辨计算机断层扫描（computed tomography, CT）进行随访，实体瘤的疗效评价标准（Response Evaluation Criteria in Solid Tumors, RECIST）为疾病进展后对患者再次行组织活检，采用突变扩增系统（amplification refractory mutation system, ARMS）法检测出*EGFR*-T790M突变组患者18例；非*EGFR*-T790M组18例，收集以上患者血清于新疆医科大学附属肿瘤医院肿瘤研究所进行保存。其中*EGFR*-T790M突变组男8例，女10例，年龄47岁-79岁，平均年龄（61.82±9.12）岁。非T790M突变组男12例，女6例，年龄42岁-83岁，平均年龄（62.17±10.53）岁。本研究通过医院伦理委员会审核通过。

### 1.2 实验材料和仪器

材料：Bradford蛋白定量试剂盒、蛋白质浓缩试剂盒（购自Bio-Rad）、TMT质量标记试剂盒和试剂、LC-MS级超纯水、LC-MS级甲酸、LC-MS级乙腈（购自Thermo）、考马斯亮兰R-250购自Amresco、二硫苏糖醇、碘乙酰胺购自Sigma、质谱级胰酶购自Promega、丙酮（购自国药）、氨水（购自Sigma）、水饱和酚（购自Solarbio）。仪器：L-3000 HPLC（购自RIGOL）、EASY-nLCTM 1200纳升级UHPLC、QExactive^TM^ HF-X质谱仪、C18除盐柱、低温离心机（购自Thermo）、恒温混匀器（购自Scilogex）、冷冻干燥机（购自Labogene）、电泳仪、电泳槽（购自Bio-Rad）、电子天平（购自Sartorius）、涡旋混合器（购自Scientific Industries）、RT-6100酶标仪（购自雷杜）、制冰机（购自雪科）、3k超滤管（购自Millipore）、组织研磨仪（购自上海净信）、超声波细胞破碎仪（购自宁波新芝）。样本收集：采取以上纳入患者晨起空腹外周静脉血5 mL，将采取标本放入（EDTA）乙二胺四乙酸二钠的抗凝管中，4, 000 r/min离心10 min，取血清储存于-80 ℃冰箱备用。

### 1.3 实验方法

#### 1.3.1 血清高丰度蛋白去除

将两组清样本混样后使用ProteoMiner蛋白质浓缩试剂盒去除高丰度蛋白，改善蛋白质检测的分辨率并对试验样品的制备进行优化。

#### 1.3.2 总蛋白提取

取出样品低温研磨成粉于液氮预冷离心管中，加裂解液后冰水浴超声5 min充分裂解。离心后取上清液加入二硫苏糖醇56 ℃反应1 h，于碘乙酰胺室温避光反应1 h。加入4倍体积的丙酮于-20 ℃条件下沉淀至少2 h，于4 ℃、12, 000 r/min离心15 min后收集沉淀。加入1 mL -20 ℃预冷丙酮重悬并清洗沉淀，同等条件下再次离心收集沉淀，风干加入适量蛋白溶解液溶解蛋白沉淀。

#### 1.3.3 蛋白质检

使用Bradford蛋白定量试剂盒，按说明配制浓度梯度为0 µg/µL-0.5 µg/µL BSA标准蛋白溶液。取不同浓度的BSA标准蛋白溶液及不同稀释倍数的待测样品加入96孔板中，补足体积至20 µL，每梯度重复3次。加入180 µL G250染色液，室温放置5 min，测定595 nm吸光度。绘制标准曲线并计算待测样品的蛋白浓度。各取20 µg蛋白待测样品进行12%SDS-PAGE凝胶电泳，浓缩胶电泳条件为80 V、20 min，分离胶电泳条件为120 V、90 min。电泳结束后行考马斯亮蓝R-250染色，脱色至条带清晰。

#### 1.3.4 TMT标记

各取120 µg蛋白样品，加蛋白溶解液补足至100 µL，加入3 µL浓度为1 µg/µL胰酶和500 µL TEAB缓冲液，混合匀后至37 ℃酶切过夜。加入等体积的1%甲酸，混匀于室温、离心取上清通过C18除盐柱，使用1 mL清洗液连续清洗3次后加入0.4 mL洗脱液洗脱2次，洗脱样合并冻干。加入100 µL TEAB缓冲液复溶，加入41 µL TMT标记试剂，室温下颠倒混匀反应2 h。终止反应后取等体积标记的样品混合，除盐冻干。

#### 1.3.5 馏分分离

配制流动相A液和B液。使用1 mL A液溶解标记混合样品粉末，室温下12, 000 r/min离心10 min，取1 mL上清进样。使用L-3000HPLC系统，色谱柱为Waters BEH C18，柱温设为50 ℃。每分钟收集1管合并为10个馏分，冻干后各加入0.1%甲酸溶解。

#### 1.3.6 高效液相色谱-质谱联

使用EASY-nLC^TM^ 1200纳升级UHPLC系统液相色谱洗脱。使用Q Exactive^TM^ HF-X质谱仪，Nanospray Flex^TM^（ESI）离子源，设定离子喷雾电压为2.3 kV，离子传输管温度为320 ℃，质谱采用数据依赖型采集模式，质谱全扫描范围为350 m/z-1, 500 m/z，一级质谱分辨率设为60, 000（200 m/z），C-trap最大容量为3×10^6^，C-trap最大注入时间为20 ms；选取全扫描中离子强度TOP 40的母离子使用高能碰撞裂解（HCD）方法碎裂，进行二级质谱检测，二级质谱分辨率设为15, 000（200 m/z），C-trap最大容量为1×10^5^，C-trap最大注入时间为45 ms，肽段碎裂碰撞能量设为32%，阈强度设为8.3×10^3^，动态排阻范围设为20 s，生成质谱检测原始数据（.raw）。

### 1.4 数据分析方法

#### 1.4.1 质谱数据分析

质谱下机数据格式为*raw，存放质谱数据完整的扫描信息，下机后的raw文件直接导入到Proteome Discoverer 2.2软件进行数据库检索，谱肽、蛋白定量, 本次使用数据库：homo_sapiens_uniprot_2019.01.18.fasta（169389 sequences）。搜库参数为：使用赛默飞超高分辨质谱仪，标记量化，酶切类型为胰蛋白酶，最大允许2个酶漏切位点，前体离子搜库时质量偏差容忍范围10 ppm，碎片离子搜库时质量偏差容忍范围0.02 Da，特定的可变修饰类型：氧化+15.995 Da（M）、TMT 10plex/+229.163 Da（K），N末端的修饰类型：乙酰化+42.011 Da、TMT 10plex/+229.163 Da，固定修饰类型：脲甲基化+57.021 Da。为了提高分析结果质量，降低假阳性率，可信度在99%以上的谱肽（peptide spectrum matches, PSMs）为可信PSMs，至少包含一个unique肽段（特有肽段）的蛋白为可信蛋白，保留可信的谱肽和蛋白，并做FDR验证，去除FDR大于1%的肽段和蛋白。

#### 1.4.2 生物信息学分析

采用GO数据库分析蛋白的细胞组分，分子功能及生物过程；基于KEGG数据库PATHWAY部分分析蛋白质参与的主要生化代谢途径和信号转导途径，使用Interproscan软件对蛋白质结构域和功能注释；利用StringDB蛋白质互作数据库进行鉴定蛋白的互作分析。

#### 1.4.3 蛋白质鉴定、定量及质控

使用Proteome Discoverer 2.2软件对检索结果过滤：保留可信度在99%以上的谱肽和蛋白，FDR时去除FDR大于1%的肽段和蛋白，鉴定出607种蛋白。搜库完成后进行肽段长度分布、母离子质量容差分布、Unique肽段数分布、蛋白覆盖度分布、蛋白分子量分布质控，蛋白可信度及整体准确性高。

#### 1.4.4 血清差异蛋白筛选

对两组蛋白质进行定量后，将每个蛋白在比较样品对中的生物重复定量值的均值的比值作为差异倍数（fold change, FC）。为了判断差异的显著性，将每个蛋白在两个比较对样品中的相对定量值进行*t*检验，并计算相应的*P*值，以此作为显著性指标。当FC≥1.0同时*P*≤0.05时蛋白表现为表达量上调，当FC≤1.00同时*P*≤0.05时蛋白表现为表达量下调。

## 2 结果

与*EGFR*-T790M基因突变相关差异性蛋白筛选：基于质谱检测得到的原始数据鉴定出共607种蛋白质，筛选出共17种差异性蛋白, 上调蛋白6种，下调蛋白11种，结果见表 1。

**表 1:** 与*EGFR*-T790M突变相关表达上调和下调的蛋白 Proteins with up-regulated and down-regulated expression associated with *EGFR*-T790M mutation

Protein	Description	Gene	log2FC	Up/Down
F2RM37	Coagulation factor Ⅸ	*F9*	0.20	Up
Q96IY4	Carboxypeptidase B2	*CPB2*	0.10	Up
A0A0B4J1V6	Immunoglobulin heavy variable 3-73	*IGHV3-73*	0.43	Up
A0A075B6R2	Immunoglobulin heavy variable 4-4	*IGHV4-4*	1.86	Up
A2N7P4	mmunoglobulin mu-chain D-J4-region	*IGHM*	0.50	Up
Q13103	Secreted phosphoprotein 24	*SPP2*	0.17	Up
Q7Z664	-	*DKFZp779N0926*	-0.25	Down
P00747	Plasminogen	*PLG*	-0.09	Down
B7Z1F8	cDNA FLJ53025 highly similar to complement C4-B	-	-0.46	
A0A140VJI7	Testicular tissue protein Li 61	-	-0.21	Down
P00742	Coagulation factor X	*F10*	-0.26	Down
Q9UGM5	Fetuin-B	*FETUB*	-0.28	Down
A0A0J9YVY3	Immunoglobulin heavy variable 7-4-1	*IGHV7-4-1*	-0.24	Down
A8K3I0	cDNA FLJ78437, highly similar to Homo sapiens cartilage oligomeric matrix protein (COMP), mRNA	-	-0.28	Down
Q8TBD0	-	-	-0.47	Down
000391	Sulfhydryl oxidase 1	*QSOX1*	-0.24	Down
A0A1W6IYJ4	N90-VRC38.03 light chain variable region (Fragment)	-	-0.79	Down
“-”: Represent Uncharacterized; EGFR: epidermal growth factor receptor.				

### 2.1 差异蛋白GO富集结果及分析

将差异蛋白数据进行GO富集分析，17种差异蛋白中参与分子功能（molecular function, MF）占40.74%，细胞组分（cellular component, CC）占7.41%，生物过程（biological process, BP）占51.85%。主要参与蛋白水解作用（proteolysis），细胞过程调节（regulation of cellular process），骨骼重塑过程（bone remodeling）等生物过程；构成细胞外区域（extracellular region）的主要成分；主要参与作用于*L*-氨基酸肽段激活肽段活性（peptidase activity acting on *L*-amino acid peptides），硫醇氧化酶活动（thioloxidase activity）等分子功能如图 1A。

**图 1:**
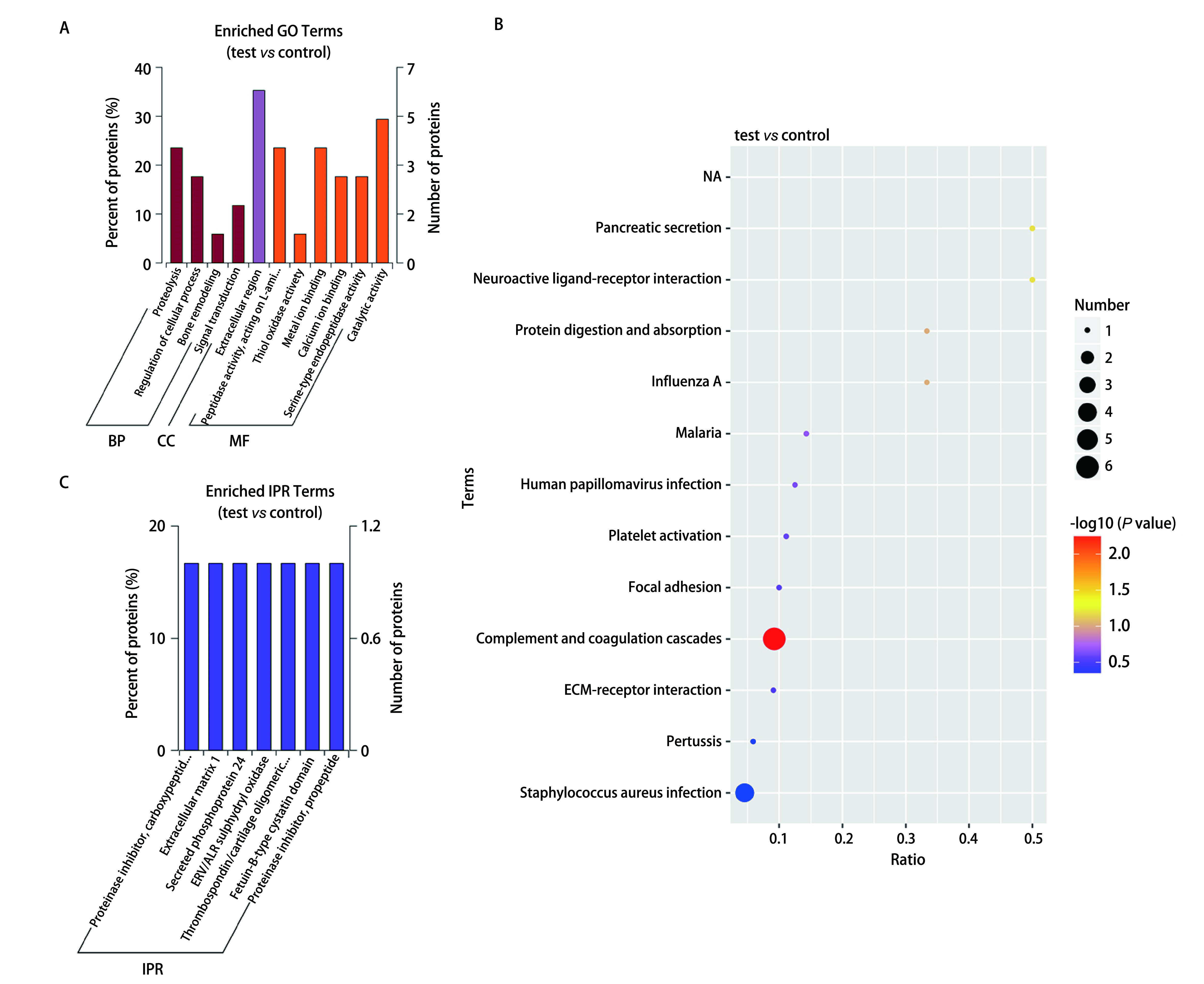
差异蛋白相关富集图表。A：差异蛋白GO功能富集结果；B：差异蛋白KEGG富集气泡图；C：差异性蛋白结构域富集柱状图。 Differential protein-related enrichment diagram. A: GO function annotation analysis of differential protein; B: KEGG enrichment scatter plot of differential protein; C: Histogram of enrichment of different protein domains.

### 2.2 差异蛋白KEGG富集结果及富集通路分析

KEGG显著性富集分析差异蛋白中显著性富集的路径。能确定差异蛋白参与的最主要生化代谢途径和信号转导途径。KEGG路径富集结果见图 1B，常见的功能条目是：疟疾（malaria）、人类乳头瘤病毒感染（human papillomavirus infection）、血小板激活（platelet activation）等。对所有差异分布蛋白的KEGG通路分析发现了12个富集通路如：血纤维蛋白溶酶原、补体和凝血级联反应、神经活性配体-受体相互作用等，途径作图显示富集指数最高的是补体和凝血级联反应，表明该途径中的关键因素可能是潜在的能预测*EGFR*-T790M耐药突变生物标志物，与该途径相关潜在生物标记物见表 2。

**表 2:** 与补体和凝血级联反应相关差异性蛋白T Different proteins associated with complement and coagulation cascade reactions

Protein ID	Protein target	Gene	log2FC	Up/Down
P00747	Plasminogen	*PLG*	-0.096, 9	Down
F2RM37	Coagulation factor Ⅸ	*F9*	0.20	Up
P00742	Coagulation factor X	*F10*	-0.257, 0	Down
Q96IY4	Carboxypeptidase B2	*CPB2*	0.101, 7	Up
Q7Z664	Uncharacterized protein	*DKFZp779N0926*	-0.254, 9	Down
B7Z1F8	Uncharacterized protein	*cDNA FLJ53025*	-0.460, 5	Down

### 2.3 差异性蛋白结构域富集分析结果

对差异性蛋白结构域富集进行分析，发现具有蛋白酶抑制剂羧肽酶前肽（proteinase inhibitor, carboxypeptidase propeptide）、细胞外基质-1（extracellular matrix 1）、磷蛋白质分泌-24（secreted phosphoprotein 24）等结构域的差异蛋白可能与肺腺癌患者发生*EGFR*-T790M耐药基因突变相关。差异性蛋白结构域富集柱状图见图 1C。

## 3 讨论

与其他不可逆*EGFR*-TKIs一样，Osimertinib与ATP结合位点中的半胱氨酸-797残基发生不可逆反应，与T790M突变体的结合力是WT *EGFR*的100倍-200倍^[1]^，因此Osimertinib是肺癌精准治疗的延续。最新FLAURA研究数据结果显示^[3]^：先前未经治疗的*EGFR*突变晚期NSCLC患者一线接受Osimertinib，中位总生存期为38.6个月，而一线使用一代EGFR-TKI患者中位总生存期为31.8个月。因此Osimertinib被批准一线用于具有*EGFR*敏感突变的晚期NSCLC患者。但因为医保问题，多数的患者仍选择在一代EGFR-TKI出现耐药并检测出*EGFR*-T790M敏感突变后使用Osimertinib。在临床实践中患者因进展病位于：脑、肝脏、骨等部位致重复活检的风险增加，而与Osimertinib失之交臂。血清中的蛋白检测可以呈现肿瘤在整个身体的情况而不是局限于某一特定位置。许多研究^[9, 10]^表明，肿瘤细胞耐药与某些蛋白高表达有关，蛋白质组学分析可用于鉴定生物标记物和评估生物网络的强大工具。信号蛋白之间的相互联系与恶性肿瘤治疗至关重要。本研究采用蛋白组学技术筛选出与*EGFR*-T790M基因突变相关的差异性蛋白：其中上调蛋白6种，下调蛋白11种，参与26种分子功能，7种细胞组分，26种生物过程。所参与通路中富集指数最高的是补体和凝血级联反应，该通路中上调的蛋白有：F2RM37（凝血因子Ⅸ）、Q96IY4（羧肽酶B2），下调的蛋白有：P00747（纤维蛋白原）、P00742（凝血因子X）、Q7Z664（未描述）和B7Z1F8（未描述）。

羧肽酶B2：是金属羧肽酶家族的成员，羧肽酶B2本身可以通过介导肿瘤微环境干扰肿瘤生长。有研究^[11-14]^表明，羧肽酶B2不仅在凝血途径中扮演重要角色，其水平显著增加乳腺癌和肺癌，胃癌和多发性骨髓瘤等的血栓发生，在乳腺癌组织、卵巢癌和肝癌细胞系中也较正常组织高表达，羧肽酶B2下调可以抑制肿瘤的侵袭和迁移。本研究中因此羧肽酶B2属于上调蛋白，有潜力成为预测*EGFR*-T790M耐药基因突变的生物标志物。

F2RM37（凝血因子Ⅸ）是维生素K依赖性凝血因子。凝血因子Ⅸ通过FⅫ触发并激活从而参与内源性凝血途径，目前在乙型血液病中被广泛研究，缺乏凝血因子Ⅸ乙型血液病病患者需终身注射FⅨ制剂^[15]^，目前尚无研究关于凝血因子Ⅸ在肿瘤发生、发展中的作用。

凝血因子X是凝血激活的重要步骤之一，可以激活内皮蛋白C受体和蛋白酶激活受体1，凝血因子X及其激活物在癌症的生长和传播中起着重要的作用。内皮蛋白C受体可在结直肠癌、肺癌、恶性胸膜间皮瘤、乳腺癌、卵巢癌、胃癌等肿瘤组织中表达增高，凝血因子X激活在胃癌进展中也起了一定的作用^[16, 17]^。另一项研究^[18]^对21例子宫内膜癌标本进行蛋白Z、蛋白Z依赖性蛋白酶抑制剂和凝血因子X进行免疫组织化学分析，发现正常子宫内膜组织均未表达以上3种因子，而在子宫内膜癌细胞中可观察到中到强度表达。凝血因子X及其激活产物在包括肺癌在内各类恶性肿瘤生长及转移中起着一定的作用，极有潜力成为预测*EGFR*-T790M耐药基因突变的生物标志物。

P00747（纤溶酶原，Plasminogen，PLG），纤溶酶原是纤溶酶的无活性前体，通过组织型纤溶酶原激活物、尿激酶纤溶酶原激活剂激活成为纤溶酶。既往有研究同样通过蛋白组学实验证实，纤溶酶原与卵巢恶性肿瘤的发生发展密切相关，血清中及组织中的纤溶酶原过表达均有潜力成为检测卵巢癌预后良好的生物标志物^[5, 19, 20]^。此外Serafin等^[21]^也发现凝血途径中的纤溶酶原激活物抑制剂-1尿纤溶酶原激活物在健康患者的血浆的平均水平为前列腺癌患者水平的8倍，可见凝血级联途径在肿瘤的发生及发展中起着及其重要的作用。

综上，本研究通过同位素标记相对和绝对定量标记结合二维液相色谱串联质谱定量蛋白组学技术共筛选出17种差异性蛋白参与各种生物过程、细胞组分、种分子功能，其中补体和凝血级联反应与*EGFR*-T790M耐药突变密切相关，而该途径中的差异蛋白：羧肽酶B2、凝血因子Ⅸ升高，凝血因子X、纤溶酶原降低均有可能成为*EGFR*-T790M耐药突变相关生物标志物，可为无法二次活检或体液检测假阴性的患者做补充，避免部分患者因以上原因错失第三代*EGFR*-TKI而致疾病持续进展。另外本研究对血清中所检测到的标志物进行初步筛选，样本量较少，后续将扩大样本量在活检组织标本中进一步证实。
